# Knowledge, Attitudes, Perceptions and Consumption of Edible Insects at the University of Venda: A Descriptive Synthesis of Three Independent Cross-Sectional Studies

**DOI:** 10.3390/insects17080868

**Published:** 2026-08-20

**Authors:** Netshivhangoni Dzulani Flexy, Ntsako Mashego, Mahlangu Sbusiso, Khavhatondwi Rinah Mofokeng, Rhulani Caswell Chauke

**Affiliations:** 1 Department of Nutrition, Faculty of Health Sciences, University of Venda, Thohoyandou 0950, South Africarhulani.chauke@univen.ac.za (R.C.C.); 2 Department of Life and Consumer Sciences, University of South Africa, Florida 1710, South Africa

**Keywords:** edible insects, entomophagy, food security, University of Venda, knowledge, attitudes, perceptions, consumption, Limpopo, mopane worm, sustainable protein, South Africa

## Abstract

**Background**: Edible insects constitute a highly nutritious, environmentally sustainable and culturally significant food resource with considerable potential to contribute to food system transformation within academic and broader societal contexts. Although global interest in entomophagy continues to increase, there remains limited research exploring the knowledge, perceptions, attitudes and consumption practices of university populations within rural South African settings. **Methods**: Three concurrent cross-sectional quantitative studies were conducted at the University of Venda (UNIVEN), located in Thohoyandou, Vhembe District, Limpopo Province, South Africa. Study 1 evaluated the knowledge, attitudes and consumption patterns of edible insects among 200 students. Study 2 examined knowledge and utilisation of edible insects among 390 students. Study 3 investigated knowledge and perceptions regarding edible insects among 200 university staff members. Structured questionnaires covering socio-demographic characteristics, knowledge, perceptions, attitudes and food frequency practices were administered across all studies. Data analysis was performed using SPSS version 28 through descriptive statistics, frequencies and percentages. Analyses were descriptive only; no inferential testing, factor analysis or regression was undertaken, and the three studies are compared as a descriptive synthesis rather than pooled into a single dataset. **Results**: Findings across all study groups demonstrated high levels of awareness regarding edible insects, with awareness reported by 85% of participants in Study 1 and 100% in Study 2, and 98% of staff members acknowleded the ecological importance of insects. Mopane worms (*Gonimbrasia belina*), termites and grasshoppers emerged as the most recognised and frequently consumed edible insect species. Consumption prevalence was notably high, reported at 91% in Study 1 and 69.2% in Study 2 and 66.3% among staff in Study 3. General attitudes towards entomophagy were largely favourable, with 41% of students expressing excitement about edible insect consumption and 91.1% of staff supporting the commercialisation of edible insects. Key barriers influencing consumption included religious restrictions (25.1%, reported in Study 2 only; participants with religious dietary prohibitions were excluded at sampling in Studies 1 and 3, limiting cross-study comparability of this barrier), cultural taboos (3%), inadequate market availability and limited awareness regarding nutritional benefits. Staff members further demonstrated positive environmental and sustainability perceptions, with 84.1% recognising edible insects as environmentally beneficial and viable future food sources. **Conclusions**: Across three independent cross-sectional studies conducted at the University of Venda, broadly similar patterns of awareness, attitudes, perceptions and edible insect consumption were observed. Although the studies differed in sampling and measurement, they consistently suggest that edible insects remain culturally accepted among many students and staff. Nevertheless, challenges relating to nutritional literacy, market accessibility and policy integration continue to limit broader adoption. Strengthening nutrition education initiatives and incorporating edible insects into institutional food systems may enhance the contribution of edible insects towards food security, sustainable protein provision and nutrition sustainability within Limpopo Province and beyond.

## Simple Summary

Insects such as mopane worms, termites and grasshoppers have been eaten for generations across southern Africa, where they provide affordable, nutritious and culturally valued food. As the world searches for healthier and more environmentally friendly ways to feed a growing population, these small creatures are attracting renewed interest. This study explored what students and staff at a rural South African university know about edible insects, how they feel about eating them and how often they do so. Combining three surveys carried out at the same university, we found that almost everyone had heard of edible insects and that many people still eat them, although staff ate them less often than students. The main barriers were not dislike but limited knowledge of the health benefits, difficulty buying insects and, for some people, religious beliefs. These findings suggest that practical steps such as offering insect-based dishes in campus dining halls and teaching people about their nutritional value could help sustain this healthy tradition. Understanding these views can guide schools, businesses and decision-makers who wish to promote edible insects as a sustainable food for the future.

## 1. Introduction

Edible insects are increasingly recognised worldwide as sustainable, nutrient-dense and environmentally sustainable alternatives to conventional animal-derived protein sources. More than 2000 edible insect species have been identified globally, with several hundred documented across the African continent and an estimated 30–50 species recorded within South Africa, many of which contain protein levels ranging from 35–75% on a dry weight basis, together with beneficial fatty acid compositions and essential micronutrients such as iron, zinc, calcium and B-vitamins that are important in addressing malnutrition, particularly within sub-Saharan Africa, where micronutrient deficiencies such as iron and zinc deficiency are more widespread than protein–energy malnutrition, and edible insects can contribute to addressing both [1,2]. In addition to their nutritional value, insect production systems require considerably lower amounts of land, water and feed compared to conventional livestock farming and produce substantially fewer greenhouse gas emissions, thereby positioning entomophagy as a promising strategy for promoting both food security and environmental sustainability [3].

Within South Africa, edible insects form an important component of traditional food systems, particularly among the vhaVenda, baPedi and Vatsonga communities of Limpopo Province. Commonly consumed species include mopane worms (*Gonimbrasia belina*), termite alates (*Macrotermes natalensis*), grasshoppers and stinkbugs (*Encosternum delegorguei*), all of which hold nutritional, cultural and economic significance within rural livelihoods [4,5]. The informal mopane worm industry alone is estimated to contribute between USD 39 and 59 million annually across southern Africa, supporting thousands of rural households, especially women involved in harvesting, processing and trade activities [6].

Despite the longstanding cultural acceptance of entomophagy in many African communities, previous studies continue to report important challenges relating to knowledge deficits, varied perceptions and socio-cultural barriers that limit the broader acceptance and formal integration of edible insects into mainstream food systems [7,8]. Factors such as limited commercial availability, religious beliefs, negative perceptions and cultural taboos continue to influence consumption practices. In this regard, university populations represent an important demographic group because students are future professionals while staff are current professionals, including policymakers, educators and healthcare practitioners, who may influence public acceptance and adoption of sustainable food practices [9]. University settings are also strategically important intervention sites, as campus cafeterias, residence catering services and curriculum integration offer concrete channels through which edible insects could be normalised and scaled. However, limited research has explored the knowledge, attitudes, perceptions and consumption (KAPC) behaviours relating to edible insects among tertiary institution communities, particularly within rural South African contexts. The present study addresses this gap through a descriptive synthesis of three concurrent studies undertaken within a single institution, providing, to the authors’ knowledge, one of the first combined KAPC profiles bridging both the student and staff strata of a rural South African university. Its contribution lies in characterising the student-to-staff gradient in entomophagy and in providing a descriptive overview against which targeted interventions can be designed and evaluated.

The University of Venda (UNIVEN), situated in Thohoyandou within the Vhembe District of Limpopo Province, provides an appropriate setting for investigating these issues. As a predominantly rural and culturally diverse institution comprising approximately 16,000 students and 7000 staff members, many originating from communities with established entomophagy traditions, UNIVEN represents a unique convergence of indigenous food practices and contemporary academic influence. This article reads three independently conducted but concurrent studies together, involving undergraduate and postgraduate students as well as academic and administrative staff members. Together, the three studies provide a descriptive profile of edible insect-related KAPC within a South African higher education context.

The study aimed to: (1) describe the socio-demographic characteristics of participating student and staff cohorts; (2) assess knowledge and awareness relating to edible insects; (3) examine attitudes and perceptions toward entomophagy, including acceptance and support for commercialisation; (4) document edible insect consumption patterns, including species consumed, preparation methods and frequency of intake and (5) identify barriers affecting edible insect consumption while proposing evidence-based recommendations for improving acceptance and utilisation.

## 2. Materials and Methods

### 2.1. Study Design and Setting

**Figure 1:**
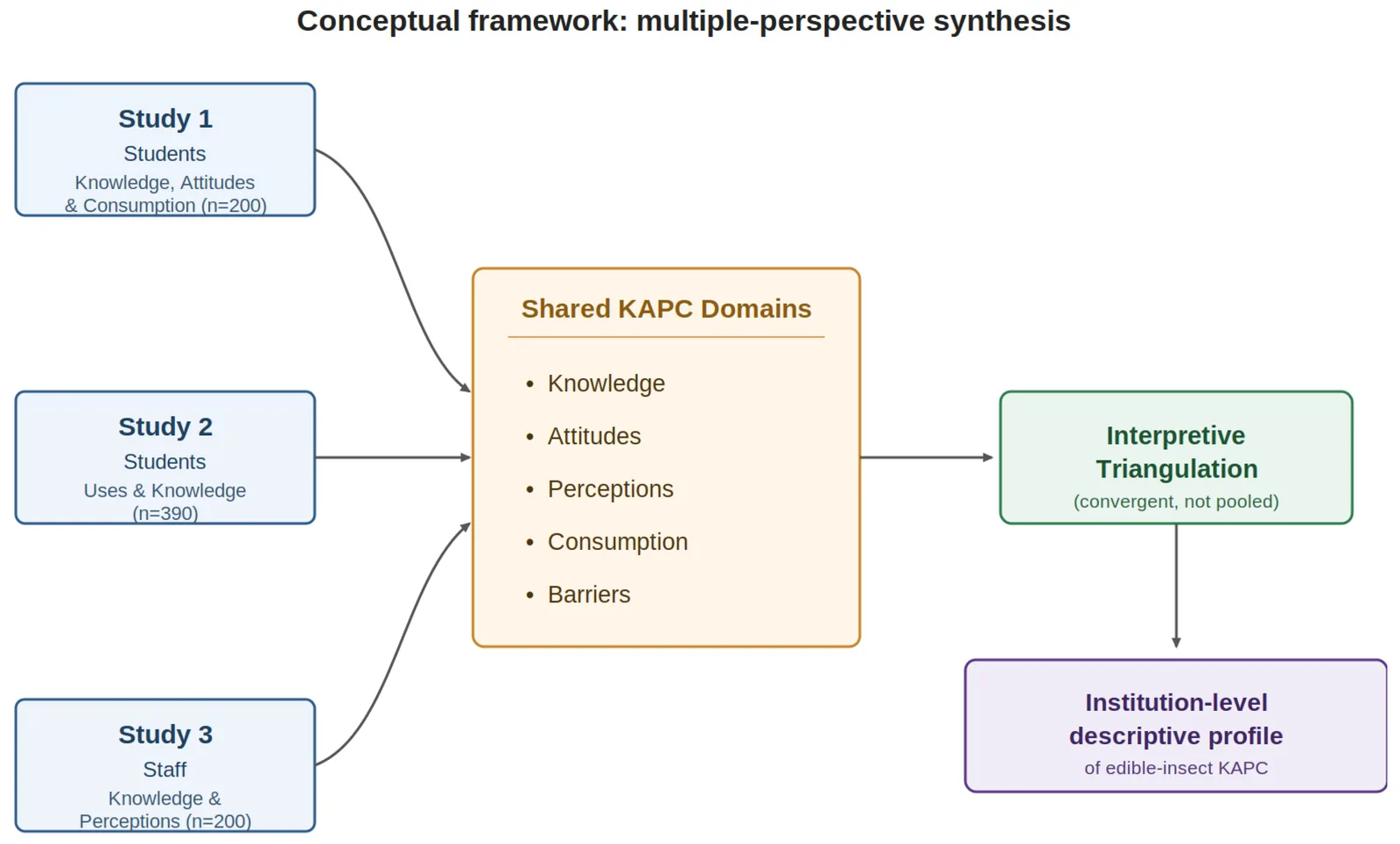
Conceptual framework for the descriptive synthesis. Three independent studies (two of students and one of staff) address an overlapping set of constructs that are mapped onto five shared KAPC domains (knowledge, attitudes, perceptions, consumption and barriers). Only conceptually equivalent, study-level summary results are compared across studies; the datasets are not pooled. A descriptive synthesis across the three studies yields an institution-level descriptive profile of edible insect knowledge, attitudes, perceptions and consumption at the University of Venda.

Three concurrent cross-sectional, quantitative studies were conducted at the University of Venda (UNIVEN), Thohoyandou, Vhembe District, Limpopo Province, South Africa (GPS: −22.9744° S, 30.4642° E). UNIVEN is a comprehensive rural-based university with four faculties, an estimated student population of 16,805 (4300 on-campus; 11,700 off-campus) and approximately 7000 staff members. The university draws its cultural strength from Venda, Tsonga and Northern Sotho traditions and has accommodated students and staff from multiple countries, including Botswana, Ghana, Kenya, Malawi, Nigeria, Swaziland and Zimbabwe. Each study employed convenience (non-probability) sampling and was conducted independently with dedicated ethical oversight, though all three followed the principles of the Declaration of Helsinki (2008) and Good Clinical Practice. The three studies were conducted and analysed independently and are brought together here as a descriptive synthesis of three parallel studies rather than as a single pooled dataset. Because each study used its own instrument, convenience sample and response categories, individual records are not comparable across studies, and no variable was re-coded to a common metric. All three studies are reported descriptively, using frequencies and percentages only; no inferential testing, factor analysis or regression was undertaken. Study-level results are placed side-by-side only where the items are conceptually equivalent, and any cross-study figures are presented as broadly indicative of the direction of findings rather than as directly comparable quantities. The overall framework is summarised in Figure 1.

### 2.2. Study 1: Knowledge, Attitudes and Consumption Among Students [10,11,12]

Study 1 targeted registered UNIVEN students (both undergraduate and postgraduate) residing on or off campus. The target population comprised 16,805 students; the sample size of 323 was determined using Slovin’s formula [*n* = N/(1 + Ne^2^)] at a 5% margin of error, and 200 students completed the survey. A structured, self-administered questionnaire was distributed in common areas including cafeterias and student residences. The questionnaire comprised three sections: (A) socio-demographics; (B) knowledge of edible insects using Likert-scale and multiple-choice items and (C) consumption using a food frequency questionnaire (FFQ)-based instrument. Students below 18 years and those belonging to religious groups prohibiting animal consumption (Jainism, Hinduism, Buddhism, vegetarianism) were excluded. Content validity was confirmed by subject matter experts (SMEs); internal consistency was assessed using Cronbach’s alpha (target ≥ 0.7). Data were analysed with SPSS Version 29. Ethical approval: FHS/24/NUT/09/0210. As only 200 of the targeted 323 questionnaires were completed and sampling was convenient, the Study 1 findings are treated as descriptive of the participating students only.

### 2.3. Study 2: Uses and Knowledge Among Students [11]

Study 2 recruited 390 registered UNIVEN students through convenience sampling at university residences and common areas. The structured questionnaire covered: (A) socio-demographic data; (B) knowledge, benefits and seasonal availability of edible insects; (C) uses, preparation methods and frequency of consumption and (D) barriers to use. Open and closed questions were used. Awareness of edible insects in Study 2 was operationalised as a single closed (yes/no) item asking whether respondents had heard of or were aware of edible insects; this item was distinct from the separate species recognition and nutritional knowledge questions and accounts for the 100% awareness figure reported below. Data were entered and analysed using SPSS version 28. Ethical approval: FHS/22/NUT/16/2303.

### 2.4. Study 3: Knowledge and Perceptions Among Staff Members [12]

Study 3 targeted UNIVEN staff members across all departments. A sample of 200 participants was recruited through convenience sampling using a semi-structured questionnaire administered through one-on-one in-depth interviews. Interviews were conducted at participants’ workplaces, recorded with a tape recorder and transcribed into English. Section A captured socio-demographics; Section B assessed knowledge (minimum 3/5 correct answers to be classified as ‘knowledgeable’), with the five items assessing recognition of edible insect species, awareness of nutritional value, seasonal availability, preparation methods and the ecological role of insects (the full instrument is available from the corresponding author). Qualitative interview responses were used to contextualise and interpret the quantitative findings reported below rather than presented as separate theme tables. Staff members from religions prohibiting insect consumption and those unavailable on data collection days were excluded. Ethical approval: FHS/22/NUT/18/0604.

### 2.5. Ethical Considerations

Informed written consent was obtained from all participants prior to data collection across all three studies. Anonymity was maintained by using participant codes instead of names. Participation was entirely voluntary, with the right to withdraw at any time without penalty. All data were stored securely with access restricted to the principal investigators. No deception or coercion was employed.

## 3. Results

### 3.1. Socio-Demographic Characteristics

Table 1 presents the socio-demographic characteristics of all three study cohorts alongside one another. In Study 1 (*n* = 200), participants were predominantly female (77%) and young adults aged 18–25 years (78%), with representation across all four university faculties. The Faculty of Sciences, Engineering and Agriculture had the highest participation (38%), followed by Health Sciences (32%). Most were undergraduates (82%), primarily in their fourth year of study (39%). In Study 2 (*n* = 390), gender was more balanced (52.6% female; 47.4% male), with a similar age distribution (76.7% aged 18–24 years). The Faculty of Health Sciences had the greatest representation (39.7%). Many students (86.4%) had financial sponsorship, and 63.3% reported a monthly income of R1000–R2000, primarily from bursaries. In Study 3 (*n* = 200), the staff cohort differed markedly: 63.4% were male, with 74.3% aged 18–34 years. Education levels ranged from Grade 12 (48.5%) to bachelor’s degree (21.8%), and reported monthly incomes ranged between R5000 and R25,000 per month. Most staff were married (86.1%).

### 3.2. Knowledge of Edible Insects

Table 2 presents knowledge findings from Studies 1 and 2. Across both studies, most participants were familiar with edible insects: 85% (Study 1) and 100% (Study 2) had heard of or knew about edible insects. In Study 1, the depth of knowledge was moderate for most students (47%), with 24% reporting very high knowledge and only 8% very low knowledge. In Study 2, 76.2% of students were aware of the nutritional benefits of edible insects, with 69.7% knowing they are nutrient-rich. Knowledge of seasonal availability was reported by 76.7% of Study 2 participants, predominantly identifying summer as the peak season for edible insect availability. In Study 3, 98% of staff affirmed the ecological importance of insects. Mopane worms were the most recognised species in both student studies (49% in Study 1; highest single-species citation in Study 2), followed by termites and grasshoppers. The culturally significant species ‘thongolifha’ (*Trinervitermes trinervoides*) was also identified by Mashego’s participants. The species recognition item used in Study 3 is not reported here as a measure of edible insect knowledge. That item listed earthworms and ants alongside insects; because earthworms are annelids and ants, although insects, are not consumed as food in this setting, the item conflated edible insects with invertebrates in general and cannot be interpreted as evidence of knowledge about edible insects. Only the separate ecological role response (98% of staff affirming the ecological importance of insects) and the species recognition recorded in the two student studies (mopane worms, termites and grasshoppers) are treated as valid indicators of edible insect recognition (see Section 4.1 and Section 4.5).

### 3.3. Attitudes and Perceptions Towards Edible Insects

Table 3 summarises attitude and perception data across all three studies. In Study 1, attitudes were largely positive: 41% of students expressed excitement about consuming edible insects, 17% were willing to try, 13% were curious and only 5% felt disgusted. Health benefits (35%) and cultural acceptance (33%) were the dominant motivators for willingness to consume, followed by taste and flavour (22%) and environmental sustainability (2%); comparable individual-level motivation data were not separately recorded for staff in Study 3. In Study 2, 69.2% of students actively used edible insects, and this behaviour was consistent with the positive attitudes observed, although the cross-sectional design cannot establish a temporal or causal relationship. In Study 3, staff perceptions were strongly positive: 84.1% agreed or strongly agreed that insects are environmentally friendly and beneficial for maintaining gas balance; 88.1% agreed or strongly agreed that insects are associated with fewer non-communicable diseases (obesity, hypertension, diabetes) and 84.1% recognised insects as a viable future food source for alleviating hunger and malnutrition. About 91.1% of staff strongly supported the commercialisation and marketing of edible insect products. In addition, 91.1% of staff regarded edible insects as part of traditional cuisine.

### 3.4. Consumption Patterns of Edible Insects

Table 4 presents the consumption data from the two student studies alongside one another. In Study 1, 91% of students consumed edible insects at some point. The majority consumed them occasionally (45%), with 16% consuming regularly and 8% frequently. Mopane worms dominated consumption (66%), followed by grasshoppers (16%), stinkbugs (2%), alates (4%) and termites (5%). About 57% of students expressed willingness to incorporate more edible insects into their diet, while 15% stated they would not and 28% remained uncertain. Education on nutritional benefits was the most cited factor that would encourage increased consumption (59%), followed by increased availability in local markets (20%). In Study 2, 69.2% of students reported current use of edible insects, primarily for consumption (66.4%) and occasionally for medicinal purposes (3.1%). The most common consumption frequency was monthly (36.7%), followed by weekly (12.8%). Frying was the preferred preparation method (41%), followed by boiling or frying (16.2%) and boiling alone (12.6%). Street vendors were the primary source from which consumers obtained edible insects.

### 3.5. Barriers to Edible Insect Consumption

Table 5 presents barriers to edible insect consumption across all three cohorts. Religious restrictions emerged as the most prominent structural barrier in Study 2, with 25.1% of students indicating that religious beliefs (including Zion Christian Church restrictions on mopane worm consumption) precluded edible insect use. Study 1 excluded participants with religious dietary prohibitions at the sampling stage. Cultural taboos were reported as a barrier by 3% of Study 2 participants. Availability and accessibility were cited by small but notable proportions in Study 2 (1% and 1.5%, respectively). Knowledge gaps, particularly regarding nutritional value and preparation methods, were identified across all three studies: 47% of Study 1 students had only moderate knowledge, 23.8% of Study 2 students were unaware of edible insect benefits and staff in Study 3 identified the absence of edible insects in the university cafeteria as a practical barrier to normalisation. Sensory concerns (taste, texture, appearance) were noted in the literature context by Study 1 and Study 3, where hesitancy around taste and preparation safety was recorded. Approximately 69% of Study 2 participants reported no barriers at all, reflecting the deep cultural integration of entomophagy in the Vhembe region.

## 4. Discussion

### 4.1. Knowledge

Study 1 showed that most students were familiar with edible insects: 85% had heard of or knew about them, although self-rated knowledge was more modest, with 47% describing their knowledge as moderate, 24% as very high and 8% as very low. Mopane worms were the most recognised species (49%), followed by termites and grasshoppers.

Study 2 similarly found high familiarity, with 100% of students affirming a single awareness item and 76.2% being aware of the nutritional benefits of edible insects; 76.7% knew about seasonal availability, mainly identifying summer. Mopane worms were again the most cited species, and the culturally significant ‘thongolifha’ (*Trinervitermes trinervoides*) was also named.

Study 3 also observed high recognition among staff, 98% of whom affirmed the ecological importance of insects. The species recognition item in Study 3, however, listed earthworms and ants alongside insects; because earthworms are annelids and not insects at all, responses to this item cannot be interpreted as knowledge about edible insects and are not analysed as such. Valid species recognition is therefore taken from the two student studies, in which mopane worms were the most recognised species, followed by termites and grasshoppers.

These patterns are broadly consistent with earlier South African work documenting extensive indigenous knowledge of edible insects and termites in the Vhembe District and beyond [4,5], indicating that the high awareness observed here reflects an established regional food culture rather than a novel finding. Comparable familiarity has been reported among student and community populations elsewhere on the continent: undergraduates in south-western Nigeria showed widespread awareness of edible insects as food [13], and studies in Ghana likewise found entomophagy to be a common, knowledge-embedded practice shaped by availability and tradition [14]. At the same time, the gap observed here between broad awareness and more limited nutritional knowledge echoes reviews emphasising that edible insects are a rich but under-appreciated source of protein and micronutrients [2,15] and that consumer understanding of their specific nutritional and food safety attributes often lags behind general familiarity [7]. This points to nutritional and food safety literacy, rather than basic awareness, as the more appropriate target for education in this setting.

Taken together, these observations suggest that awareness of edible insects was high in all three groups, consistent with the entomophagous tradition of Limpopo Province, but that the depth of nutritional knowledge appears more limited than bare awareness, a gap also noted in the wider literature, where public awareness has tended to outpace detailed nutritional knowledge.

### 4.2. Attitudes and Perceptions

Study 1 recorded largely positive attitudes: 41% of students were excited about eating edible insects, 17% were willing to try and 13% were curious, while only 5% expressed disgust. The main motivators were health benefits (35%) and cultural acceptance (33%), with taste (22%) and environmental sustainability (2%) less prominent.

Study 2 similarly pointed to acceptance, with 69.2% of students reporting that they actively used edible insects; this is consistent with positive attitudes, though the cross-sectional design cannot establish a temporal or causal link.

Study 3 also observed strongly positive perceptions among staff: 84.1% agreed that insects are environmentally friendly, 88.1% associated them with fewer non-communicable diseases and 84.1% saw them as a viable future food source. Support for commercialisation was high (91.1%), and 91.1% regarded edible insects as part of traditional cuisine.

The strongly favourable attitudes and low disgust observed across all three cohorts contrast sharply with findings from Western consumer research, where eating insects is typically a novel behaviour. In European and other high-income settings, willingness to consume insects is frequently constrained by disgust, food neophobia and limited familiarity: profiling studies identify only a minority of consumers as ready to adopt insects as a meat substitute [16], acceptance in the Netherlands and Italy is shaped heavily by prior exposure and product framing [17,18], and food neophobia and disgust sensitivity are consistently among the strongest negative predictors of the intention to eat insect-based foods [19,20,21]. The 5% disgust recorded in Study 1 therefore most plausibly reflects the lifelong cultural exposure of this population, placing the University of Venda cohorts nearer the acceptance end of the global spectrum than the neophobia-dominated Western samples. The predominance of health- and culture-based motivations over environmental ones also differs from Western studies, in which sustainability arguments feature prominently in acceptance [21,22]; where insects are already a familiar food, framing them as traditional and nutritious may be more persuasive than sustainability messaging.

One possible interpretation is that favourable attitudes were common across all three groups, with the low 5% disgust in Study 1 well below rates often reported in Western settings and in keeping with the idea that cultural exposure supports acceptance. Motivations appeared more nutritional and cultural than environmental, which fits a setting where insects are a familiar food rather than a novel intervention. The high staff support for commercialisation is the most policy-relevant observation, but it rests on a single-item, cross-sectional measure and is best treated as hypothesis-generating rather than established.

### 4.3. Consumption

Study 1 showed that 91% of students had eaten edible insects at some point, most of them occasionally (45%), with 16% consuming them regularly and 8% frequently. Mopane worms dominated consumption (66%) and 57% of students were willing to eat more, with education on nutritional benefits the most cited encouragement (59%).

Study 2 similarly found substantial use, with 69.2% of students currently using edible insects. Monthly consumption was most common (36.7%), frying was the preferred preparation (41%) and street vendors (33.6%) had overtaken bush harvesting (24.1%) as the main source.

Study 3 also observed consumption among staff, with 66.3% reporting some intake, at 21.8% monthly, 16.8% weekly and 27.7% annually, and cooking (28%), roasting (21.8%) and drying (14.9%) as the main preparation methods.

The continued, everyday consumption observed here aligns with evidence that entomophagy remains an economically and culturally embedded practice in the region rather than a declining one. The dominance of mopane worms and the shift toward vendor-based sourcing in Study 2 are consistent with the documented commercialisation of the mopane worm in rural Limpopo, where harvesting, processing and trade sustain substantial informal livelihoods [6,23]. The preparation methods reported, principally frying, roasting, boiling and drying, mirror the traditional and increasingly value-added processing techniques described for edible insects across Africa [24], while the occasional medicinal use noted in Study 2 reflects the well-documented role of insects in traditional food and medicine on the continent [25]. Comparable consumption patterns, driven by availability, taste and tradition, have been reported among student and community groups in Ghana and Nigeria [13,14], reinforcing the interpretation that the practice at the University of Venda forms part of a wider, living regional and continental food system.

Taken together, these observations suggest that eating edible insects remains a living practice in all three groups rather than a residual tradition, with mopane worms recurring as the dominant species. The shift toward vendor-based sourcing in Study 2 mirrors the region’s wider commercialisation trend. Consumption appeared somewhat lower among staff than students, though the different frequency categories used across studies make this a descriptive difference rather than a tested contrast.

### 4.4. Barriers

In Study 1, participants whose religion prohibits the relevant foods were excluded at the sampling stage, so religious barriers could not be observed; the main barrier noted was a knowledge gap, with 47% of students reporting only moderate knowledge, alongside sensory concerns drawn from the literature.

Study 2, which did not apply this exclusion, found religion to be the most commonly reported barrier (25.1%, including Zion Christian Church restrictions on mopane worm consumption), followed by cultural taboos (3%) and small proportions citing availability (1%) and accessibility (1.5%); notably, about 69% of participants reported no barriers at all, and 23.8% were unaware of the benefits.

Study 3 also excluded religiously restricted staff at sampling; here, the practical barriers noted were the absence of edible insects from the university cafeteria and some hesitancy around taste and preparation safety.

The barrier profile identified here is only partly comparable to that reported elsewhere, and the differences are themselves informative. In Western consumer studies, the dominant obstacles are psychological, principally disgust and food neophobia [19,20], whereas in the present setting, the leading measured barrier was religious restriction, most notably Zion Christian Church prohibitions on mopane worm consumption, a structural and belief-based barrier that is largely absent from the international literature. Food safety and quality concerns, a recurring theme in reviews of edible insect consumption [7], appeared here mainly as hesitancy around taste and preparation safety. Importantly, the recurring knowledge gap and the absence of edible insects from institutional catering are practical, modifiable barriers rather than fixed cultural ones; this accords with foresight and food systems analyses arguing that mainstreaming edible insects depends less on overcoming outright rejection than on improving availability, information and integration into everyday and institutional food environments [9,26,27]. Engaging religious leaders, closing nutritional knowledge gaps and introducing edible insects into campus catering therefore emerge as the most actionable levers in this context.

Taken together, these observations suggest that the barrier picture differs across the studies partly by design: because Studies 1 and 3 excluded religiously restricted participants, the 25.1% religious restriction figure applies to Study 2 only. Knowledge gaps recur in all three, the Zion Christian Church restriction stands out as an under-reported barrier for this region and the absence of edible insects from institutional catering emerges as a practical, potentially addressable barrier.

### 4.5. Strengths and Limitations

The main strength of this work is that it brings together three concurrent studies of students and staff at a single institution, giving a broad descriptive picture of edible insect knowledge, attitudes, perceptions and consumption in a rural South African university. Several limitations should be weighed against this. The three studies were independent and used different instruments and response categories, so cross-study figures are broadly indicative rather than directly comparable. All three relied on convenience sampling, which limits generalisability beyond the University of Venda. The Study 3 staff cohort was drawn mainly from younger support, service and administrative personnel (74.3% aged 18–34 years) rather than senior academic staff, so it describes this subgroup rather than the workforce. Comparison of barriers is constrained because religiously restricted participants were excluded at sampling in Studies 1 and 3. Finally, the work is wholly descriptive: no inferential testing, factor analysis or regression was possible, because the studies were held as separate datasets with no harmonised participant-level file. Future work would benefit from a single harmonised, probability-sampled instrument, allowing for analysis of outcomes by sex and other factors and appropriate inferential modelling of the associations that the present descriptive design can only describe.

## 5. Conclusions

Across three independent cross-sectional studies conducted within the same university, broadly similar patterns emerged. Awareness of edible insects was high in every group, ranging from 85% in Study 1 to 100% on the single awareness item in Study 2, attitudes were generally positive and a substantial share of participants reported eating edible insects, from 66.3% among staff to 91% among students. Mopane worms were consistently the most recognised and most consumed species. Because the three studies used separate instruments and convenience samples, these figures are best read as three separate findings that point in a similar direction rather than as a single pooled estimate for the university.

The studies also point, separately but consistently, to a few recurring gaps. In each group, awareness appeared broader than detailed nutritional knowledge. Religious restriction was a notable barrier where it was measured (25.1% in Study 2, including Zion Christian Church restrictions), although it could not be estimated in the other two studies, which excluded religiously restricted participants at sampling. Market access for campus-based students rested largely on informal vendors, and edible insects were absent from institutional catering.

These observations point to, rather than establish, some possible directions: nutrition education that goes beyond basic awareness; exploring the inclusion of edible insects in campus catering; engaging religious community leaders in any promotion and attention to product quality and safety. Because the evidence is descriptive and drawn from three convenience samples at a single institution, these are best treated as hypotheses for further work rather than firm recommendations. The modest contribution of reading the three studies together is to show that similar patterns recur across separate student and staff samples at one rural South African university, a starting point for the harmonised, probability-based and inferential research that would be needed to confirm them.

## Figures and Tables

**Table 1:** Consolidated socio-demographic characteristics of student and staff participants at the University of Venda, Limpopo Province, South Africa.

Characteristic	Students (Netshivhangoni; *n* = 200)	Students (Mashego; *n* = 390)	Staff (Mahlangu; *n* = 200)
Gender: Female (%)	77	52.6	34.7
Gender: Male (%)	23	47.4	63.4 (male majority)
Age: Majority group	18–25 yrs (78%)	18–24 yrs (76.7%)	18–34 yrs (74.3%)
Employed/income	N/A (students)	5.4% employed; 63.3% earn R1000–R2000/month	R5000–R25,000/month
Highest faculty/dept (*n*, %)	Sciences, Eng. and Agric. (38%)	Health Sciences (39.7%)	Grade 12 = 48.5%; Bachelors = 21.8%
Sample determination	Slovin’s formula; *n* = 323 targeted; 200 completed	Convenience sampling; *n* = 390	Convenience sampling; *n* = 200

Note: EI = edible insects; N/A = not assessed in that study. Study 1 = Netshivhangoni [10]; Study 2 = Mashego [11]; Study 3 = Mahlangu [12]. The Study 3 staff sample was drawn predominantly from younger support, service and administrative personnel (74.3% aged 18–34 years; 86.1% married) rather than from senior academic staff, and its findings should therefore not be generalised to the university workforce (see Section 4.5).

**Table 2:** Knowledge of edible insects among University of Venda students (Studies 1 and 2).

Variable	Students: Netshivhangoni (*n* = 200) *n* (%)	Students: Mashego (*n* = 390) *n* (%)
Aware of edible insects	170 (85%)	390 (100%)
Moderate/average knowledge level	94 (47%)	N/A (all aware; varied depth)
High/very high knowledge	80 (40%)	~69.7% knew nutritional value
Most recognised species—Mopane worm	98 (49%)	19.2% listed mopane worm among multiple species
Grasshoppers recognised	20 (10%)	12.1%
Termites recognised	30 (15%)	7.7%; also noted with thongolifha
Know seasonal availability	Not assessed	76.7%; mainly summer
Know benefits of edible insects	Not directly scored	297 (76.2%); ‘rich in nutrients’

Note: EI = edible insects. Study 1 = Netshivhangoni [10] (*n* = 200); Study 2 = Mashego [11] (*n* = 390). Staff knowledge (Study 3) reported narratively (see Section 3.4). Species recognition percentages are not directly comparable between the two studies: Study 1 recorded each respondent’s single most-recognised species, whereas Study 2 permitted the listing of multiple species, so the mopane worm figures (49% in Study 1 versus the leading single-species citation in Study 2) reflect different question formats. “Awareness” in Study 2 (100%) refers to a single closed item asking whether respondents had heard of or were aware of edible insects and is distinct from the species recognition and nutritional knowledge items.

**Table 3:** Attitudes and perceptions towards edible insects across student and staff cohorts at the University of Venda.

Statement/Variable	Students: Netshivhangoni (*n* = 200)	Students: Mashego (*n* = 390)	Staff: Mahlangu (*n* = 200)
Positive/excited about edible insects	41% excited; 17% willing to try; 13% curious	69.2% use EI; positive overall	91.1% perceive EI as traditional food
Disgusted/negative	5%	Religion/culture as barrier (25% and 3%)	None strongly disagreed with EI nutritional value
EI environmentally friendly	2% cited environmental sustainability as motivation	Not directly asked	84.1% agree/strongly agree
EI as future food source	Not directly assessed	Not directly assessed	84.1% agree/strongly agree
Support commercialisation of EI	Not directly assessed	Not directly assessed	91.1% strongly support
Health concern reduction (obesity, diabetes)	35% cited health benefits as motivation	Not directly asked	88.1% agree/strongly agree
Cultural acceptance as motivator	33%	Culture = 3% barrier; acceptance generally high	N/A—framed as traditional food

Note: EI = edible insects. Study 1 = Netshivhangoni [10] (*n* = 200); Study 2 = Mashego [11] (*n* = 390); Study 3 = Mahlangu [12] (*n* = 200).

**Table 4:** Edible insect consumption patterns among University of Venda students (Studies 1 and 2).

Consumption Variable	Students: Netshivhangoni (*n* = 200) *n* (%)	Students: Mashego (*n* = 390) *n* (%)
Ever consumed EI	182 (91%)	270 (69.2%)
Consume occasionally	90 (45%)	Monthly: 141 (36.7%)
Consume regularly/weekly	32 (16%)	Weekly: 50 (12.8%)
Consume frequently/yearly	16 (8%)	Yearly: 21 (5.3%)
Primary species: Mopane worm	132 (66%)	19.2% (single species); highest combined prevalence
Grasshoppers consumed	32 (16%)	12.1%
Termites consumed	10 (5%)	7.7% (termites alone)
Preferred preparation: Frying	Not separately reported	160 (41%)
Main purchase/harvest source: Street vendors	Not directly assessed	131 (33.6%); bushes 94 (24.1%)
Would incorporate more EI into diet	114 (57%)	Not directly assessed
Key encouragement: Education on nutritional benefits	118 (59%)	25.9% primary reason for consumption: nutrition

Note: EI = edible insects. Study 1 = Netshivhangoni [10] (*n* = 200); Study 2 = Mashego [11] (*n* = 390). Staff consumption (Study 3) reported narratively in Section 3.4.

**Table 5:** Barriers to edible insect consumption across student and staff cohorts at the University of Venda.

Barrier Category	Students: Netshivhangoni (*n* = 200) *n* (%)	Students: Mashego (*n* = 390) *n* (%)	Staff: Mahlangu (*n* = 200) *n* (%)
Religion	Excluded (Jainism/Hindu/Buddhist/vegetarian)	98 (25.1%)	EI-prohibiting religion: excluded
Cultural norms/taboos	Not separately reported	12 (3%)	Significant for older demographics
Availability/accessibility	Not reported	Availability: 4 (1%); Accessibility: 6 (1.5%)	Not in university cafeteria (qualitative)
Taste/sensory discomfort	Negative sensory perception noted in literature review	Not directly reported	Hesitancy on taste and safety noted
Limited knowledge/awareness	Knowledge gap identified (47% moderate)	23.8% unaware of benefits	2% unaware of ecological role
No barriers reported	N/A	270 (69%)	N/A

Note: EI = edible insects. Study 1 = Netshivhangoni [10] (*n* = 200); Study 2 = Mashego [11] (*n* = 390); Study 3 = Mahlangu [12] (*n* = 200).

## Data Availability

Data are available on request from the corresponding author, subject to privacy and ethical restrictions.
